# Assessing the perceived value of a user‐led educational intervention to support recovery in a Swedish psychiatric organization: A qualitative case study

**DOI:** 10.1111/hex.14064

**Published:** 2024-05-02

**Authors:** Maria Reinius, Lina Al‐Adili, Inka Helispää Rodriguez, Terese Stenfors, Mats Brommels

**Affiliations:** ^1^ Department of Learning, Informatics, Management and Ethics, Medical Management Centre Karolinska Institutet Stockholm Sweden; ^2^ North Stockholm Psychiatry Health Care Services Stockholm County Stockholm Sweden; ^3^ Division for Learning, Department of Learning, Informatics, Management and Ethics Karolinska Institutet Stockholm Sweden

**Keywords:** coping, educational intervention, empowerment, mental health, Patient School, Recovery College, stigma reduction

## Abstract

**Introduction:**

Many people with mental health issues recover and re‐establish their identity and find hope and meaning in life, irrespective of symptom burden. Recovery can be supported through learning and education, aiming at strengthening self‐management and coping skills. Such education offered by peers with lived experience is rare and scarcely reported. The aim was to assess the perceived value of an educational intervention, called the Patient School (PS), organized within a psychiatry organization by employed patient peers with lived experience.

**Methods:**

A qualitative case study based on interviews with people with mental health issues (*n* = 8), peer‐organizers (*n* = 4) and healthcare professionals (*n* = 4), and documents such as schedules and educational materials were used. First, the interviews were transcribed and analyzed using inductive conventional content analysis. Second, the findings were synthesized into a programme theory, illustrated in a logic model.

**Results:**

The perceived value of the PS was related to the willingness of peer‐organizers to share their own experiences, a sense of belonging, sharing with like‐minded and new knowledge, practical skills, roles and attitudes acquired. These experiences were empowering, decreased stigma and reassured user participants that one's identity is not defined by mental health issues. This increased self‐confidence paves the way for increased self‐management and creates a potential for a more efficient use of healthcare services.

**Conclusion:**

We conclude that this PS, organized within a psychiatry organization by salaried peers, achieved the same positive results as those reported in the literature and showed the value of having peer‐organizers being part of the staff.

**Patient or Public Contribution:**

This research was performed in a partnership between academic researchers and persons with user experience of psychiatric services, engaged in the educational intervention in the focus of the study. The research plan was co‐designed, and the analysis of the data collected was performed in collaboration. The participation of the co‐researchers with user experience gave the project team access to the study site, provided the team with insights into to study context and contributed with an understanding promoting the interpretation of the findings.

## 1 INTRODUCTION

Many people with mental illness recover and there are several routes towards recovery, whereof medical treatment is one of many.^1^, ^2^ Key components in recovery may be to re‐establish one's identity and to find hope and meaning in life, and build a satisfying life as one self‐define it, irrespective of ongoing or recurring symptoms.^3^ This can be achieved by shifting focus from problems and dysfunctions, and instead supporting people to identify and develop their talent and skills, explore new possibilities and strive towards realizing their goals.^4^ It has, consequently, also been described as an ‘assets‐based’ approach, aiming at developing the ‘recovery capital’ of patients, defined as ‘the array of social, psychological and cultural networks beyond professional inputs’.^5^

One of several methods that have been suggested to enhance recovery is education.^6^, ^7^ Lifelong learning has been found to improve well‐being and enhance recovery in mental illness,^8^ and improve social networks.^9^ However, discrimination and stigmatization often exclude people with mental illness from accessing education.^7^

One prominent example of this educational approach is the network of ‘recovery colleges’ in the United Kingdom. Presently coordinated by the network ‘Implementing Recovery through Organisational Change’. Around 40 Recovery Colleges (RCs) engage over 500 peer workers promoting learning and self‐management as core practices amongst mental health patients,^10^ and entailing skills and employment training. These learning activities are characterized by their co‐production, developed and organized by peers with experience of mental illness and healthcare professionals. While RCs have been evaluated in research, few of those evaluations have been co‐produced.^11^ Chalmers and Glasziou have demonstrated that researchers need to change their ways of working to improve the relevance of research to patients and involve patients in the research process.^12^ Incorporating the perspectives of peers with experience of mental illness in research might provide new insights.^11^

A qualitative study on long‐term effects found that course participants expressed gains related to intrapersonal changes, increased opportunities and ethos of recovery and equality that sustained at 1 year follow‐up after attending courses at RC.^13^ The Patient School (PS) was inspired by and has much in common with RCs although it is shorter and less resource‐intensive. A recent systematic review concluded that ‘RC [recovery college] attendance was associated with high satisfaction among students, attainment of recovery goals, changes in service providers' practice, and reductions in service use and cost’.^14^ Another review found that participants felt that attending an RC was useful and enhanced progress towards recovery goals—there were also indications of reductions in service use.^15^

The focus of the current study is a patient‐driven educational intervention in Sweden named the *PS*. The intervention is implemented and developed by persons working with user involvement and organizational development in a psychiatric care organization. These positions require personal experience of recovering from mental illness.

The Stockholm PS research is part of the ‘Patient in the Driver's Seat’ programme, featuring patient‐led innovations.^16^ The creation of the programme was a collaborative effort with a patient innovator who not only co‐developed the programme's aims and methodologies but also assisted in assembling the management team, including four other patient innovators. This team worked in tandem with researchers to direct the programme's execution.

The context of the study is a Swedish psychiatry organization providing both inpatient and outpatient care. The PS was started in 2018 by persons with lived experience, working within the organization as ‘user involvement coordinators (UIC)’ or ‘staff with user experience (SUC)’. The school is organized in facilities within the psychiatry organization with the support of its leadership and coordinated by employed UICs and SUCs. In total, 12 courses were given with close to 70 course participants. The PS consists of a series of five workshops given over 5 weeks. The course leaders invited healthcare professionals from the psychiatry organization or researchers to act as co‐leaders and substance matter experts.

The PS was initiated by UICs within the psychiatry sector to enhance user involvement and offer supplemental peer support. Drawing from the recovery movement and their own experiences, they provided practical information about mental health services while respecting the individuality of each patient's journey and the importance of evidence‐based practices. The programme's design was influenced by a study visits to UK RCs, but it was adapted to meet local needs.

The overall aim of our co‐produced research was to perform a detailed study of the PS and explore how course participants, course leaders and invited experts describe it in terms of what it is and what values it brings. To add an explanatory approach, we synthesized the findings into a programme theory of the PS, illustrated as a logic model.^17^, ^18^ We used lived experience involvement in the co‐design and analysis of the study to gain a deeper understanding of the value of the PS, guiding future implementations and studies in similar contexts.

## 2 MATERIALS AND METHODS

This is a qualitative case study of the PS, involving the participation of researchers and persons with user experience in both the design and execution of the research.^12^ To ensure the quality and transparency of this research, we have adhered to the Consolidated Criteria for Reporting Qualitative Studies guidelines^19^ (Supporting Information S1: File 1).

Course leaders informed participants of the study. Invitations to participate in interviews were sent by one of the authors M. R., introduced as a researcher interested in understanding participants' experiences within the PS. Invitations were sent to participants of the PS who had shared their contact information during or after completing the programme (*n* = 45), healthcare professionals who participated as experts (*n* = 7) and course leaders (*n* = 6). No relationship was established before study commencement, except for one person who is part of the Research Group International Relations. The numbers of those who responded positively and the time when interviews were performed are exhibited in Table 1.

**Table 1:** Workshop themes, content and focus areas within the Patient School.

Theme	Content and focus
Psychiatry—how does it work?	Understanding the roles and the assistance available
Recovery—what is helpful?	Effective strategies and support systems that have facilitated the recovery process for individuals overcoming severe mental illness, based on their own experiences
Other resources in the society	Outlines the practical assistance and processes offered by various community organizations in support of individuals
Relations and disclosure	Addresses strategies for discussing personal well‐being with family, friends and colleagues, and discuss the extent of openness that is appropriate in various relationships
Personal tools	Explores various self‐assistance techniques and tools individuals can utilize for self‐care. It also examines the effectiveness of these methods based on the experiences of others who have employed them

All interviewees, referred to as participants, received written information about the study in advance and were able to ask questions before the interview started. The Patient Consent Form, was collaboratively developed through the [Patients in the driver's seat programme], involving patients and researchers. Their informed consent was recorded and included in the transcriptions of all interviews. Due to the nature of this research, participants of this study did not agree for their data to be shared publicly, and participant characteristics is not provided to minimize the risk of identification.

A semistructured interview guide was developed by the research team, focusing on participants' perceived values of the PS, including open‐ended questions, such as: *how would you describe the purpose of the PS? What value do you think the PS creates? For patients? For staff? For the healthcare organization? Is there anything about the PS that has been particularly valuable to you*? and probes.

The interviews were conducted over the telephone by M. R. at the researcher's office and were recorded and transcribed verbatim by a professional transcriber. M. R. with expertise in qualitative research methods and interviewing techniques, guided interviewees to openly share their reflections, employing probes with minimal interference. Data collection ended when no further relevant information on the investigated topic was obtained from the interviews.

Course leaders provided documents related to the PS, such as educational material, advertisements, user information and other documents that they found relevant for the study. Course leaders also informed the research team about how the PS had been conceptualized, their ambitions and overarching goals. In total, eight‐course participants, four healthcare professionals and four course leaders participated in interviews lasting on average 37 min (between 25 and 75 min) (Table 2).

**Table 2:** Timeline of respondent recruitment.

Time period	Group of respondents invited	Method for invitation	*N* respondents invited	*N* respondents interviewed
March 2021	Participants who had participated in previous PSs (2018–2020)	Email	39	5
April 2021	Healthcare professionals who had participated in previous PSs (2018–2020)	Email	6	3
May 2021	Participants at ongoing PSs	M. R. visited the recovery college	6	3
May 2021	Healthcare professionals who participated in ongoing PSs	M. R. visited the recovery college	1	1
May 2021	Leaders of previous and ongoing PSs	Email	6	4

The interview transcripts were analyzed with conventional content analysis, following each step thoroughly. First, M. R. read through all transcripts several times to reach immersion and wrote condensed meaning units to all sections of the text that corresponded to the aim. M. B. read five randomly selected transcripts and identified meaning units well corresponding to those proposed by M. R. The condensed meaning units without any reference to participants were subsequently transferred to post‐it notes on an online Miro board.^20^ Through a series of three workshops attended by all authors, categories were identified, defined, and coded. M. R. and L. A.‐A. selected direct citations from the interviews to illustrate each category. All authors reviewed the initial findings and suggested revisions until a consensus was reached.

During the analysis process, a representation of the views of the involved stakeholders on how programme inputs and activities can be linked to intended and observed outcomes evolved, thus forming a tentative causal model or programme theory of the PS.^18^ This empirical pattern is presented as a logic model with six components in a logical sequence—target population, assumptions, programme inputs, activities, outputs and outcomes.^16^ The input of the UIC team members had a substantial impact on how the results were interpreted. Wordings in interviews were put into the organizational context and further explained, for instance, by describing in what situation those probably had been expressed. This greatly increased the understanding of the team members academically educated and helped in formulating the tentative explanations. All team members participated in discussing and revising this manuscript, a first draft of which was written by the member who had performed the interviews. The authors have combined expertise encompassing qualitative research, user experience and professional practice of psychiatric care. In qualitative research, the researcher functions as the key instrument for data collection, analysis and interpretation.^21^ The study's methodology is thus shaped by the authors' diverse backgrounds and its co‐designed approach.^11^ Continuous and thorough discussions amongst all authors enriched the data analysis.

## 3 RESULTS

The synthesis of our findings according to Hayes' logic model is exhibited in Table 3, followed by condensations of our observations with direct quotes from participants.

**Table 3:** A logic model of the Patient School (PS).

Target population	Assumptions	Inputs	Activities	Outputs	Outcomes
People who use mental health services	—People with mental health issues can recover and the PS can be supportive on the path towards recovery Assumptions regarding course leaders —Course leaders need to have own experience of mental illness Assumptions regarding healthcare personnel —Are willing to participate and understand the aim and method of the PS Assumptions regarding participants —People who use mental health services are interested in participating in the PS	Resources Course participants Course leaders with own experience of mental illness Healthcare professionals willing to participate as invited experts Help with recruitment and advertisement Rooms Written material that course participants can keep	One course with a standardized package of 5 workshops over 5 weeks. The aim is to learn together about recovery and how resources within psychiatric care and society can be used Course participants, course leaders and healthcare personnel learn from each other Each workshop has a theme: 1. Psychiatry—how does it work? 2. Recovery—what is helpful? 3. Other resources in society. 4. Relations and disclosure 5. Personal tools.	Course participants develop knowledge and skills A sense of belonging and sharing with like‐minded Course leaders share their own experience An opportunity to take on other roles and meet each other in other roles	Empowerment—participants go from passive recipients of care to active partners Decreased stigma—participants feel supported in that the illness is not their identity Efficient use of healthcare resources

### 3.1 Target population and assumptions

The PS targets individuals with mental health issues, guided by some underlining assumptions described in the assessed documents. The central assumption is that people with mental health issues can recover, and that the PS has a supportive role in facilitating this process.

A course leader articulated the overarching goal as follows; So, the broader purpose is to, to … foster or enable recovery. It […] is about health, and living a life that one … feels one possesses or has control over… So, it's much more about empowerment and health and quality of life, I would say. Then about whether one is healthy or sick. (Participant 13)

Three assumptions concerning the three stakeholder groups were highlighted: course leader possesses own experience of mental illness which enables them to connect with participants of the PS. One course leader noted:

When participants find out that one is experienced oneself, it becomes easier to start talking about oneself. Because they know that the course leaders are also experienced themselves. I believe it opens, it actually opens many channels, of personal experience. (Participant 14)

Healthcare professionals are willing to participate and understand the aim and method of the PS, which ensures an efficient collaboration. People who use mental health services are interested in participating in the PS and are committed to supporting their own recovery and health.

### 3.2 Programme input and activities

The PS relies on resources for its success, including course participants, course leaders who have personal experience with mental illness and healthcare professionals who are willing to contribute as invited experts and help with recruitment and advertisement. In addition to human resources, the programme uses physical resources such as dedicated rooms and educational materials that participants can keep.

The PS consists of one course comprising a standardized package of five workshops over 5 weeks. The main aim is to learn together about recovery and how resources within psychiatric care and society can be used. Central to the programme is sharing experience and learning from each other amongst stakeholders. Each workshop has a different focus, covering the following themes: Psychiatry—how does it work?; Recovery—what is helpful?; other resources in the society; relations and disclosure and personal tools (described in Table 1).

### 3.3 Outputs

#### 3.3.1 Knowledge and skills

Course participants expressed gaining knowledge about their needs in the PS and how they could get help to address them. They highlighted that clear information was provided with an overarching explanation of what support was available for people with mental illness, and how to get access to it.

Healthcare professionals described that participants could learn from a patient perspective, something that staff may have limited knowledge about. Both course participants and course leaders said that the PS focused on human beings, in contrast to psychiatric care that was described as focusing on medicine, symptoms and problems.

Even though people have different diagnoses, at the PS, it was very rewarding that … people meet as fellow humans in having some kind of difficulty. And how they have tackled it. So, it was a very pleasant atmosphere, sharing different experiences with each other. And that, just that, which I thought was good, was that those who … were mainly in charge, that they had experience with mental illness. Then you know that they know what it's about. Even though everyone has different experiences then. (Participant 15)

Course participants described gaining new perspectives, like understanding that they themselves could take responsibility for their care and that they could ask for help. Course leaders also thought that the course participants became more aware of their rights.

#### 3.3.2 A sense of belonging and sharing with like‐minded

Both course participants and course leaders saw value in meeting other people with similar experiences. Course participants said that the PS made them feel less lonely and course leaders acknowledged that this was one of the purposes with the PS. One course participant described getting a sense of togetherness and said:

You felt a sense of togetherness with everyone, that you belonged and that you were a little bit the same […] that you are not alone in everything that happens, and that there are other people that doesn't know either. (Participant 3)

Course participants noticed that they were not alone with their questions, and that it was valuable to hear other peoples' thoughts and experiences about how they reasoned, for example, about relationships and when and how they could be open about their condition. Course participants also said that they felt comforted by seeing that other people also struggle but at the same time have a good life.

Course participants said that they felt trusted and listened to, to a larger extent than they did in a care planning meeting with staff. Health professionals also described the PS as a forum where course participants could feel safe and contrasted this to care meetings where patients may not have time to think and come up with questions.

#### 3.3.3 Course leaders share their own experiences

Course participants expressed that it was valuable that the course leaders were open with their own experiences of psychiatric illness, of being a patient and recovery. Course participants said that they trusted what the course leaders said and that it felt good to talk to someone who had similar experiences but had come further towards recovery. The course leaders could explain how the healthcare system worked in relation to relevant authorities. Their experience could be trusted; they did not try to ‘trick them’. The course leaders had a sense for how participants felt and could spontaneously bring up topics that were important to them but that no one dared to raise. Course leaders expressed similar thoughts and said that it was important to share their experiences with the course participants. The varying experience of course leaders helped them to identify complementing content to include in the workshops. However, course leaders emphasized that the medical information they give is evidence‐based, a natural stance given their employment status. That information though, it was said, could be easier for participants to relate to when it was delivered by someone with personal experience. In some cases, course leaders provided information that they thought that staff did not know was important for participants. For example, course leaders presented an overview of all resources in the community available to people with mental health issues. Course leaders expressed that only those who have user experience could understand the importance of such an overview.

Both course participants and course leaders expressed that the mix of peer leaders and health professionals as lecturers was good as they could deliver complementary information. Course leaders and course participants expressed that the overview of care and authorities provided by the course leaders only could be presented by persons who had experience of being a patient in these systems. Course leaders highlighted that they, with personal experience, often gave the same information as staff but that course participants perceived their messages differently.

#### 3.3.4 Opportunity for meeting each other in different roles

Course leaders described that health professionals could take a more personal role when they visited the PS, felt as a safe space, compared to their usual professional role.

You perceive the health care personnel as being more personal than usual. (Participant 1)

In contrast, this opportunity was not mentioned by staff, and answering a direct question they said that they had the same role in the PS as when they met patients in the care setting. Course leaders emphasized that staff could gain new perspectives by participating in the PS where they meet patients who have recovered in contrast to only meeting patients when they are acutely ill.

### 3.4 Outcomes

#### 3.4.1 The patient goes from a passive recipient of care to an active partner

Course participants said that they felt empowered after the PS and course leaders expressed hope that the PS would make the course participants more involved in their care and feel that they had power over their own life. Staff also said that they thought that the PS would make patients feel safer in having influence over their own care and they described that patients who had participated in the PS could be bolder and ask more questions. As an example, one course participant said that she would focus the next meeting with her nurse towards more long‐term issues and not just discuss the present condition. When asked if the PS would change anything in relation to staff, the course participant said:

Yes, I think I will use this to structure future meetings so that the meetings with the nurse is not just about how I feel right now, but more with a long‐term perspective. (Participant 4)

#### 3.4.2 Decreased stigma

Course leaders said that the main purpose of the PS was to change the way patients think about themselves, to strengthen healthy behaviours and to reduce stigma related to mental illness. Staff confirmed this by saying that they had perceived the purpose of the PS to be to normalize mental illness and reduce stigma.

So, the benefit is that … we experience it, those of us who hold these, various topics that concern all participants in some way, and that one can get answers to their questions. And I also believe that this results in reduced stigma, the stigmatization around your own illness. So, one discovers that just like me, you don't need to feel bad because there are others who feel the same way. And participants share experiences among themselves. But I believe a lot in the issue of stigmatization, prejudices, that it decreases among the participants after completing the training. (Participant 6)

Course participants did not use the word stigma but described that people could have prejudices towards people with mental illness and that they felt stronger, after the course, that the illness was not their identity. One course participant said that talking to peers helped to disregard prejudice that one could meet in society.

I notice that some have prejudice about people with psychiatric disease […] and before I took it badly and I took it personally. But know that I went to this education I feel stronger. And it is like a protection to know that I don't have to tell others. (Participant 5)

#### 3.4.3 Efficient use of healthcare resources

Course leaders described that course participants had the potential to use healthcare resources more efficiently.

They use what is available to them in a smarter way and they know where to turn and what they can get […] Patients that have more information about what is available can use that in another way. They can ask for what they think that they need […] and go directly to that service without waiting to get called. (Participant 2)

As the course participants learn what health care can offer and who does what, they can request the help that they think they need, instead of waiting for a meeting where they can describe their problem to staff and hoping for a response. Course leaders also said that course participants' expectations about what they could get from health care could change from participating. For example, some course participants could be disappointed because it was difficult to get access to therapy, but in the PS they learned that others got helped by talking to a mental nurse aid.

## 4 DISCUSSION

We have presented a case of an educational intervention for persons with mental health issues, organized by peers, and resembling a UK RC, using a logic model representing its programme theory by identifying assumptions, resources used, and activities organized and providing a tentative explanation of those outputs and outcomes as results of those activities.

The central *assumptions* were that people with mental health issues can recover, that peer learning can support that process, and that both service users and providers are willing to participate in those learning activities. Those individuals constitute the most important *resources* in addition to physical ones. The PS was organized as a series of workshops covering different themes (*activities*).

Interviewees described the value of participating in the PS as follows: the willingness of course leaders to share their own experiences, a sense of belonging and possibility to share with like‐minded, knowledge and practical skills acquired, and the opportunity to identify and experience new roles and behaviours, being tangible *outputs* of the PS. These experiences were empowering and enabled the transition from passive recipient of care to active partner, decreased feeling of stigma and developed a sense that one's identity is not defined by the mental health issue. These *outcomes* were seen to have the potential to lead to a more efficient use of available healthcare services.

That persons with user experience were responsible for planning and managing the five workshops was central to the success of the PS. Their activities demonstrated the value of peer support, the importance of which is widely acknowledged. Mahlke et al.^22^ highlighted in a review article the need for clarification of roles and competencies to ensure benefits to receivers of peer support. The literature also supports that peer support providers should be included in the mental health workforce.^23^ These success factors were present in our case, as the PS leaders were employed by the psychiatry department as user‐involvement coordinators or staff with user experience, and were provided resources, training and support from senior management. Hence, RCs within psychiatric settings might foster a cultural transformation towards more holistic care, promote mutual learning and advocate for the importance of hope, empowerment and community integration in the recovery journey.

Course participants stressed the importance of belonging to a community of peers, the knowledge and skills they had acquired and how that made them feel empowered. These are important elements of coping with everyday challenges and contribute to the process of recovery. Positive coping is associated with lower levels of distress and higher personal well‐being.^24^ Two previous reviews of the literature identified similar recovery processes amongst people with mental health issues. They were connectedness (demonstrated as rewarding interpersonal relationships and social inclusion), hope and optimism about the future, rebuilding positive identities, finding meaning in life and empowerment.^25^, ^26^ By employing those mechanisms, the PS could contribute to positive coping and recovery amongst its participants. Interviewees mentioned more efficient use of healthcare resources as one outcome of the PS. Empirical studies seem to confirm such a possibility. A follow‐up study of participants in a UK RC reported a decline in mental health service use. Reductions in hospital bed days, admissions and community contacts were statistically significant.^27^

PS participants emphasized how their positive experiences had led to a decreased feeling of stigma. There is a vast literature on how the social environment can contribute to decreasing stigma, which, indeed, is important as stigma is a barrier to community integration and recovery.^28^ However, there are fewer studies on how people with mental health issues can cope with stigma. In one of those, Prior^29^ analyzed interviews with students seeking counselling for psychological problems. When they were helped to normalize their difficulties as ordinary problems of adolescence their levels of distress were reduced, their self‐reliance grew, the threshold to seek help was lowered and they felt more confident to solve their problems. The PS seemed to trigger the same kind of ‘normalisation processes’ amongst participants and might explain their sense of decreased stigma. That these strategies and others are introduced by people with lived experience (as in our case) has further positive effects.^30^ The PS might support organizational change by driving psychiatric staff to adopt recovery‐oriented practices and recognize the value of peer‐led education and service user involvement. This represents a shift, challenging conventional perceptions of mental illness and prioritizing holistic recovery models over symptom management only. The impact of such a transformation extends beyond individual benefits and might foster a more inclusive and empowering care within psychiatric organizations. To understand the implications and effectiveness of this approach, further research is needed.

A survey study reported improvements in wellbeing and personal resources, high satisfaction with a service perceived as accepting and enabling, as well as a greater sense of hope, confidence and aspirations after attending an RC.^31^ RC participants expressed in focus group interviews that they had experienced a positive impact on their lives and seen benefits to the organization organizing the college.^32^ A review of 44 studies explored outcomes on mental health staff, service and societal levels: Staff involved in RCs experienced and valued co‐production, they appreciated changed perceptions of service users and reported increased passion and job motivation. RCs are not part of the formal care organization (even when being embedded) which allows them to develop an alternative culture, creating experiential learning opportunities for mental health professionals to realize the opportunities related to co‐production and peer workforce. At the societal level RCs expose agencies in the community and their staff to service users and offer collaboration with those with a positive impact on attitudes amongst staff and public opinion.^33^

The PS offers an approach that challenges and extends beyond traditional psychiatric standards, and aligns with outcomes at service and societal levels, such as improved staff attitudes, the development of a co‐production culture within services, and enhanced community engagement and attitudes towards mental health, described in the 5‐year programme of RC research in England.^34^ Our case from Sweden seems to confirm those positive results of RCs in United Kingdom,^35^ and the broader literature on the mechanisms behind those results is comparable to the programme theory of the PS as constructed from our empirical data, planning documents, programmes, course material and stakeholder interviews. Our ability to conceptualize these findings is a strength of the study. This was supported by a detailed understanding of the context, including activities by key actors and their relationships that were conveyed to the whole group by researchers with lived experience. The inclusion of diverse participant groups enriches our findings, presenting a spectrum of experiences and viewpoints that enhance the study's validity and contribute to a deeper understanding of the subject and the detailed context understanding provided by researchers with lived experience. Their insights have been invaluable, offering a grounded perspective that enriches the entire research. However, a weakness is the small scale of the case, but a detailed analysis of the context and its integration as boundary conditions into a programme theory, corroborated by empirical evidence from elsewhere, offer advice on a successful implementation of a PS in other environments. Such studies on the scalability of the RC concept would be an important future line of research.

## 5 CONCLUSION

The PS in Sweden, organized within a psychiatry organization by salaried peers, inspired by RCs in the UK, achieved similar positive results as reported in previous formal evaluations of RCs. Findings show the value of having peer‐organizers being part of the formal staff in psychiatric care and leading educational interventions to support individuals in their recovery. Additional research is needed to enable comparisons of outcomes of such interventions across different contexts.

## AUTHOR CONTRIBUTIONS

**Maria Reinius**: Methodology; formal analysis; conceptualization; investigation; validation; software; data curation; writing—review and editing; writing—original draft; visualization. **Lina Al‐Adili**: Writing—review and editing; writing—original draft; conceptualization; investigation; validation; methodology; formal analysis; visualization; data curation. **Inka Helispää Rodriguez**: Conceptualization; investigation; methodology; validation; visualization; data curation; writing—review and editing; formal analysis. **Terese Stenfors**: Conceptualization; investigation; writing—review and editing; validation; visualization; methodology; formal analysis. **Mats Brommels**: Supervision; writing—review and editing; funding acquisition; investigation; conceptualization; methodology; validation; visualization; formal analysis; project administration.

## CONFLICT OF INTEREST STATEMENT

The authors declare no conflict of interest.

## ETHICS STATEMENT

Ethical approval has been granted by The Regional Ethical Review Board of Stockholm (Dnr 2019‐03849 with amendment Dnr 2020‐04604). All procedures followed were in accordance with the ethical standards of the responsible committee on human experimentation (institutional and national) and with the Helsinki Declaration of 1975, as revised in 2000. Informed consent was obtained from all patients for being included in the study.

## Supporting information

Supporting information.

## Data Availability

Due to the nature of this research, interviewees of this study did not agree for their data to be shared publicly. All documents used are freely available.

## References

[1] Bracken P , Thomas P , Timimi S , et al. Psychiatry beyond the current paradigm. Br J Psychiatry. 2012;201(6):430‐434. 10.1192/bjp.bp.112.109447 23209088

[2] Slade M , Longden E . Empirical evidence about recovery and mental health. BMC Psychiatry. 2015;15(1):285. 10.1186/s12888-015-0678-4 26573691 PMC4647297

[3] Andresen R , Oades L , Caputi P . The experience of recovery from Schizophrenia: towards an empirically validated stage model. Aust N Z J Psychiatry. 2003;37(5):586‐594. 10.1046/j.1440-1614.2003.01234.x 14511087

[4] Perkins R , Pepper J , Rinaldi M , Brown H . Briefing Papers 1. Recovery Colleges. ImROC; 2012.

[5] Burns H . *Health in Scotland 2010: Assets for Health. Annual Report of the Chief Medical Officer 2010*. The Scottish Government; 2011.

[6] Perkins R , Meddings S , Williams S , Repper J . Recovery Colleges 10 Years on. ImROC; 2018.

[7] Stenfors‐Hayes T , Griffiths C , Ogunleye J . Lifelong learning for all? Policies, barriers and practical reality for a socially excluded group. Int J Lifelong Educ. 2008;27(6):625‐640. 10.1080/02601370802408274

[8] Hammond C . Impacts of lifelong learning upon emotional resilience, psychological and mental health: fieldwork evidence. Oxford Rev Educ. 2004;30(4):551‐568. 10.1080/0305498042000303008

[9] Preston J , Hammond C . Practitioner views on the wider benefits of further education. J Further Higher Educ. 2003;27(2):211‐222. 10.1080/0309877032000065226

[10] ImROC . ImROC. 2022. Accessed December 1, 2023. https://Imroc.Org

[11] Lin E , Harris H , Black G , et al. Evaluating recovery colleges: a co‐created scoping review. J Ment Health. 2023;32(4):813‐834. 10.1080/09638237.2022.2140788 36345859

[12] Chalmers I , Glasziou P . Avoidable waste in the production and reporting of research evidence. Lancet. 2009;374(9683):86‐89. 10.1016/S0140-6736(09)60329-9 19525005

[13] Thompson H , Simonds L , Barr S , Meddings S . Recovery colleges: long‐term impact and mechanisms of change. Mental Health Soc Inclusion. 2021;25(3):232‐242. 10.1108/MHSI-01-2021-0002

[14] Thériault J , Lord M‐M , Briand C , Piat M , Meddings S . Recovery Colleges after a decade of research: a literature review. Psychiatr Serv. 2020;71(9):928‐940. 10.1176/appi.ps.201900352 32460684

[15] Meddings S , McGregor J , Roeg W , Shepherd G . Recovery colleges: quality and outcomes. Mental Health Soc Inclusion. 2015;19(4):212‐221. 10.1108/MHSI-08-2015-0035

[16] Karolinska Institutet . Patients in the driver's seat! A multimethod partnership program on patient‐driven innovations. 2022. Accessed February 15, 2024. https://Ki.Se/En/Lime/Patients-in-the-Drivers-Seat-a-Multimethod-Partnership-Program-on-Patient-Driven-Innovations

[17] Hayes H , Parchman ML , Howard R . A logic model framework for evaluation and planning in a Primary Care Practice‐based Research Network (PBRN). J Am Board Fam Med. 2011;24(5):576‐582. 10.3122/jabfm.2011.05.110043 21900441 PMC3266837

[18] Rogers PJ . Using programme theory to evaluate complicated and complex aspects of interventions. Evaluation. 2008;14(1):29‐48. 10.1177/1356389007084674

[19] Booth A , Hannes K , Harden A , Noyes J , Harris J , Tong A . COREQ (Consolidated Criteria for Reporting Qualitative Studies). In: Moher D , Altman DG , Schulz KF , Simera I , Wager E , eds. Guidelines for Reporting Health Research: A User's Manual. John Wiley & Sons Ltd., 214‐226. 10.1002/9781118715598.ch21

[20] Miro . Where teams get work done. 2022. Accessed January 10, 2023. https://Miro.Com.

[21] Braun V , Clarke V . Reflecting on reflexive thematic analysis. Qual Res Sport Exerc Health. 2019;11(4):589‐597. 10.1080/2159676X.2019.1628806

[22] Mahlke CI , Krämer UM , Becker T , Bock T . Peer support in mental health services. Curr Opin Psychiatry. 2014;27(4):276‐281. 10.1097/YCO.0000000000000074 24852058

[23] Shalaby RAH , Agyapong VIO . Peer support in mental health: literature review. JMIR Ment Health. 2020;7(6):e15572. 10.2196/15572 32357127 PMC7312261

[24] Meng X , D'Arcy C . Coping strategies and distress reduction in psychological well‐being? A structural equation modelling analysis using a national population sample. Epidemiol Psychiatr Sci. 2016;25(4):370‐383. 10.1017/S2045796015000505 26077164 PMC7137609

[25] Leamy M , Bird V , Boutillier CL , Williams J , Slade M . Conceptual framework for personal recovery in mental health: systematic review and narrative synthesis. Br J Psychiatry. 2011;199(6):445‐452. 10.1192/bjp.bp.110.083733 22130746

[26] Tew J , Ramon S , Slade M , Bird V , Melton J , le Boutillier C . Social factors and recovery from mental health difficulties: a review of the evidence. Br J Soc Work. 2012;42(3):443‐460. 10.1093/bjsw/bcr076

[27] Bourne P , Meddings S , Whittington A . An evaluation of service use outcomes in a Recovery College. J Ment Health. 2017;27(4):359‐366. 10.1080/09638237.2017.1417557 29275749

[28] Roe D , Lysaker PH , Yanos PT . Overcoming stigma. In: Corrigan PW , ed. The Stigma of Disease and Disability: Understanding Causes and Overcoming Injustices. American Psychological Association; 2014:269‐282. 10.1037/14297-014

[29] Prior S . Overcoming stigma: how young people position themselves as counselling service users. Sociol Health Illn. 2012;34(5):697‐713. 10.1111/j.1467-9566.2011.01430.x 22026466

[30] Shahwan S , Goh CMJ , Tan GTH , Ong WJ , Chong SA , Subramaniam M . Strategies to reduce mental illness stigma: perspectives of people with lived experience and caregivers. Int J Environ Res Public Health. 2022;19(3):1632. 10.3390/ijerph19031632 35162655 PMC8835394

[31] Ebrahim S , Glascott A , Mayer H , Gair E . Recovery Colleges; how effective are they? J Mental Health TraiN Educ Pract. 2018;13(4):209‐218. 10.1108/JMHTEP-09-2017-0056

[32] Zabel E , Donegan G , Lawrence K , French P . Exploring the impact of the recovery academy: a qualitative study of Recovery College experiences. J Mental Health Train Educ Pract. 2016;11(3):162‐171. 10.1108/JMHTEP-12-2015-0052

[33] Crowther A , Taylor A , Toney R , et al. The impact of Recovery Colleges on mental health staff, services and society. Epidemiol Psychiatr Sci. 2019;28(5):481‐488. 10.1017/S204579601800063X 30348246 PMC6998922

[34] Hayes D , Henderson C , Bakolis I , et al. Recovery Colleges Characterisation and Testing in England (RECOLLECT): rationale and protocol. BMC Psychiatry. 2022;22(1):627. 10.1186/s12888-022-04253-y 36153488 PMC9509550

[35] Toney R , Elton D , Munday E , et al. Mechanisms of action and outcomes for students in Recovery Colleges. Psychiatr Serv. 2018;69(12):1222‐1229. 10.1176/appi.ps.201800283 30220242

