# Supplementary material for: Assessing the perceived value of a user‐led educational intervention to support recovery in a Swedish psychiatric organization: A qualitative case study
Source: Health Expect. 2024 May 2;27(3):e14064. doi: 10.1111/hex.14064 (PMC11064016; doi:10.1111/hex.14064)
Supplement: Supplementary file 1 — Supporting information. [file HEX-27-e14064-s001.pdf]

COREQ Checklist

No	Item	Guide questions/description
1. Page 5	Interviewer/facilitator	Which author/s conducted the interview or focus group?
2. Is found in author details	Credentials	What were the researcher's credentials? *E.g. PhD, MD*
3. Also found in author details	Occupation	What was their occupation at the time of the study?
4. Female and male names are found in author details	Gender	Was the researcher male or female?
5. Page 4	Experience and training	What experience or training did the researcher have?
Relationship with participants		
6. No relationship was established prior to commencement except for one person who is part of the research group (IR). See page 5	Relationship established	Was a relationship established prior to study commencement?
7. Page 5	Participant knowledge of the interviewer	What did the participants know about the researcher? e*.g. personal goals, reasons for doing the research*
8. Page 5	Interviewer characteristics	What characteristics were reported about the interviewer/facilitator? e.g. *Bias, assumptions, reasons and interests in the research topic*
**Domain 2: study design**		
Theoretical framework		
9. page 6	Methodological orientation and Theory	What methodological orientation was stated to underpin the study? *e.g. grounded theory, discourse analysis, ethnography, phenomenology, content analysis*
Participant selection		

No	Item	Guide questions/description
10. see page 5	Sampling	How were participants selected? *e.g. purposive, convenience, consecutive, snowball*
11. Page 5	Method of approach	How were participants approached? e.*g. face-to-face, telephone, mail, email*
12. Page 5	Sample size	How many participants were in the study?
13.No one refused or dropped out. Those who showed interest participated.	Non-participation	How many people refused to participate or dropped out? Reasons?
Setting		
14. page 5	Setting of data collection	Where was the data collected? e.*g. home, clinic, workplace*
15. No, only the researchers and participants<br>Page 5	Presence of non-participants	Was anyone else present besides the participants and researchers?
16. page 5-6	Description of sample	What are the important characteristics of the sample? *e.g. demographic data, date*
Data collection		
17. Page 5	Interview guide	Were questions, prompts, guides provided by the authors? Was it pilot tested?
18. No repeat interviews were carried out.	Repeat interviews	Were repeat interviews carried out? If yes, how many?
19. Page 5, audio	Audio/visual recording	Did the research use audio or visual recording to collect the data?
20. yes	Field notes	Were field notes made during and/or after the interview or focus group?
21. Page 5	Duration	What was the duration of the interviews or focus group?
22. Page 5	Data saturation	Was data saturation discussed?

No	Item	Guide questions/description
23. No	Transcripts returned	Were transcripts returned to participants for comment and/or correction?
**Domain 3: analysis and findings**		
Data analysis		
24. only the first author who conducted the interviews	Number of data coders	How many data coders coded the data?
25. no but this can be provided if requested	Description of the coding tree	Did authors provide a description of the coding tree?
26. Page 6-7	Derivation of themes	Were themes identified in advance or derived from the data?
27. Page 7	Software	What software, if applicable, was used to manage the data?
28. No	Participant checking	Did participants provide feedback on the findings?
Reporting		
29. Yes, in findings section page 7-13	Quotations presented	Were participant quotations presented to illustrate the themes / findings? Was each quotation identified? e.*g. participant number*
30. Yes, see findings section page 7-13	Data and findings consistent	Was there consistency between the data presented and the findings?
31. Yes, see findings section page 7-13	Clarity of major themes	Were major themes clearly presented in the findings?
32. Yes, see findings section page 7-13	Clarity of minor themes	Is there a description of diverse cases or discussion of minor themes?
